# Abdominal pain and fever in young women. Advantages of abdominal ultrasound in the EF

**DOI:** 10.1186/2036-7902-6-S1-A8

**Published:** 2014-01-31

**Authors:** A Oviedo-García, M Algaba-Montes, D Nuñez-Hospital, J Lopez-Libano, JM Alvarez-Franco, N Diaz-Rodriguez, A Rodriguez-Lorenzo

**Affiliations:** 1 Emergency Department, Valme Hospital, Seville, Members of the Working Group of Ultrasound SEMES_Andalucía and SEMERGEN, Spain; 2 Emergency Department, Valme Hospital. Seville, Spain; 3 Critical Care Department, Miramar Hospital, Mallorca, Member of the Working Group of Ultrasound SEMERGEN, Spain; 4 Emergency Department, IB-Salut, Ibiza, Member of the Working Group of Ultrasound SEMERGEN, Spain; 5 Primary Care. Barbadás Primary Care Center. Ourense, Member of the Working Group of Ultrasound SEMERGEN, Spain; 6 Radiology Department. Perpetuo Socorro Hospital, Vigo, Member of the Working Group of Ultrasound SEMERGEN, Spain

## Background

Liver abscess (LA) is an entity with high morbidity and mortality, and therefore require a diagnosis agile and dynamic, allowing appropriate management to avoid complications. The emergency ultrasound (US) allows a versatile and comprehensive management, improving the prognosis of this disease in the majority of cases.

## Objective

we present a case of LA, diagnosed at ED, through the use of US scanning used by Emergency Phisicians.

## Patients and methods

a patient with abdominal pain, with a final diagnosis of a EC assessing US, performed by EP.

## Results

a 20 year old woman with no history of relevance, came to the emergency room with fever of 1 month duration and abdominal pain that persists after analgesic treatment. On arrival had malaise, hypotensive, febrile, tachycardic to 110 sqm, oriented in all three spheres, with no cardiorespiratory examination findings. Nontender abdomen, without peritonitis. The rest of the examination was irrelevant. Laboratory tests showed leukocytosis and neutrophilia, and abdominal and chest radiography was usual. She underwent a bedside abdominal ultrasound, que injury showed a complex, heterogeneous, poorly defined right hepatic lobe inside anechoic and echogenic materials, 70 x 85 mm, compatible with liver abscess at this level and confirmed by abdominal CT. Developments after starting antibiotic therapy after early crops, and subsequent percutaneous drainage thereof, was satisfactory, with total resolution, remaining asymptomatic.

## Conclusion

Liver abscesses are rare pathologies, but lethal without prompt diagnosis and treatment, based this on antibiotics and percutaneous drainage after culture. Bedside abdominal ultrasound means applying an extraordinary advance in the diagnosis and evaluation of emergency physicians, being transcendental training of this technique for quality care, comprehensive and dynamic services. Its use and distribution must be paramount, since it represents a cost-effective measure quality front as shown in the case that concerns us.
